# A study on the relationship between personality and motivation in leisure participants

**DOI:** 10.1371/journal.pone.0318168

**Published:** 2025-02-19

**Authors:** Gül Yağar, Gamze Deryahanoğlu, Emine Bal

**Affiliations:** 1 Faculty of Sports Sciences, Hitit University, Corum, Türkiye; 2 Faculty of Sports Sciences, Tokat Gaziosmanpaşa University, Tokat, Türkiye; University of Tartu, ESTONIA

## Abstract

Motivation and personality, which are among the most important effects of human behavior, are important in terms of leisure activities. Therefore, this study aimed to examine the relationship between motivation and personality of individuals who participate in physical activity in their leisure time. In the study, the correlational screening model was used, and 370 (*m*:*28*.*76±10*.*37*) participants who regularly practiced physical activity participated. The relationship between the data obtained in the research was tested with canonical correlation analysis, and the first two canonical correlation functions between the two variable data sets were interpreted. Self-control and extraversion sub-dimensions in the personality data set and internal regulation and external regulation sub-dimensions in the motivation data set were found to make the highest contribution. It was determined that for the first set of canonical functions, the relationship between self-control and internal regulation had a unidirectional correlation, whereas, for the second set of canonical functions, the relationship between extraversion and external regulation had an inverse correlation. Studies show how valuable intrinsic motivation sources are for individuals, and personality traits such as self-control and extraversion support this situation. It is considered that the personality traits of leisure time participants may be a clue to the types of motivation of the participants.

## Introduction

Individuals who spend their lives between work and daily chores struggle to make time for themselves and want to spend their time with creative and quality activities [1]. Torkildsen [2] defines leisure time as activities that individuals freely choose, that are valuable to them, and that play an essential role in their self-actualization. In the broadest sense, leisure activities are an antidote to alienated labor [3] and vary across time, culture, and place [4]. When the studies on leisure activities that positively affect human life [5] and even have a relationship with overcoming problems in society [6] were examined, it was determined that the reasons affecting the motivation to participate in these activities were psychological, biological, physiological, sociological, and behavioral [7].

When the leisure literature is considered, it is seen that the sense of freedom and intrinsic motivation have a dominant aspect [8]. Motivation, which is described as the driving force and inspiration that pushes people to behave [9], varies from person to person by showing individual differences in terms of its intensity since it has a unitary structure [10]. Personality is defined as the impression that individuals leave on others [11]. When previous studies were examined, it was found that personality traits can be both stable and changeable throughout life [12]. Emotional and mental elements, which are known to be very effective in the motivation process, also affect the state of being motivated. In other words, in addition to social needs such as social roles, qualitative characteristics of social relations, belonging, and dignity, our physiological needs such as hunger and thirst are also effective in our motivation process [13]. Therefore, many internal and external factors such as our characteristics, professional life, personal experiences, and social structure affect our motivation level [14]. As Roberts and Yoon [15] state, motivation, the second domain of personality, focuses on what people consciously or unconsciously want.

Self-determination theory, one of the motivation theories, relates personality and motivation to each other and states that there are 3 types of motivation: amotivation, intrinsic motivation, and extrinsic motivation. These types of motivation are among the effective factors in revealing human behavior [16]. Moreover, according to the self-determination theory, self-determination is the state of believing oneself sufficient to meet one’s needs, believing in oneself, and having the belief that one has the power, knowledge, and skills to perform the relevant task [17].

### Personality and motivation in self-determination theory

Self-determination theory was developed by two psychologists, Edward L. Deci and Richard M. Ryan, in the late 1970s, and the basic principles of the theory were included in the 1985 book [18]. The theory is a meta-theory of individuals’ motivation and personality development [19], and addresses human behavior in areas of life including work, human relations, religion, sports, health, stereotyping, and prejudice [20]. Deci and Ryan [21] questioned the extent to which individuals actually decide on the behaviors they exhibit in terms of freedom and self-determination, and while focusing on the importance of intrinsic motivation in self-determination theory, they also stated that extrinsic motivation factors should not be ignored, and they actually mentioned that motivation is the energy that the organism needs. Self-determination theory has become an influential theory with a wealth of research evidence, providing blueprints for understanding the motivational bases of personality and social behavior and the relationship of basic psychological needs to well-being, psychological progress, and high quality of life [22].

In participating in physical activity, Fogg and Euchner [23] stated that a person must be motivated, have the ability to achieve the goal, and be motivated to perform the action. According to self-determination theory; the individual’s motivation has a wide area where extrinsic and intrinsic motivation factors are effective from the amotivation state, and as the level of internalization of motivation increases, the individual’s amotivation state changes towards intrinsic motivation [24].

Considering that individuals participate in leisure time activities with intrinsic motivation and that their personalities are also effective, this study aimed to examine the relationship between personality and motivation of leisure time participants based on self-determination theory.

## Materials and methods

### Research model

The correlational screening model was used to reveal the relationship between two or more variables. The correlational screening model aims to provide researchers with a clue about the connection between variables, rather than the cause-and-effect output between variables [25]. In line with the purpose of the study, correlation, one of the two existing types of correlational screening, was used.

### Study group (universe-sample)

The study group consisted of 370 (28.76±10.368) individuals who visited fitness centers in their leisure time. We used the purposive random sampling method due to its reliability and purposeful selection of the data [26]. According to Friday and Leah [27], purposive sampling is the preferred method for enhancing the reliability of the findings. This study, focusing on motivation, favored a mixed purposive sampling technique among other purposive sampling techniques. We preferred this technique. We favored this technique over other purposive sampling techniques because it aimed to establish the relationship between the personality and motivation types of individuals who engage in sports freely. The researchers also favored the mixed-purpose sampling technique for its flexibility in the sample process. According to the study, individuals who visit fitness centers to engage in physical activity during their leisure time make up the study’s population. Since it is not possible to determine the universe of individuals who exercise in fitness centers in their leisure time in Çorum province and to give a clear number as a result, an estimated number has been determined by the researchers. Based on the estimated sample size tables from Bartlett et al. [28] and Yazıcıoğlu and Erdoğan [29], 357 was found to be the smallest number that this estimated sample size of 5000 would cover with a 0.05 percent error. Therefore, we determined that 357 was the lowest number of participants targeted for the study.

### Procedure, and ethics

The study was approved by Hitit University Non-Interventional Ethics Committee with the decision numbered 2023-154/2023-12. After receiving ethics committee approval for the study, permission was obtained from the fitness center managers to reach the participants. Study data were collected between August and December 2023. This study collected data in accordance with the 1964 Helsinki Declaration. During the data collection process, participants were informed about the study by showing the Ethics Committee Approval of the study. Participants were informed that the information obtained during the study would be kept confidential and the results of the study would be conducted for scientific purposes only. During the data collection process, individuals were informed that they would participate in the study voluntarily if they completed the survey and that incomplete surveys would not be included in the study. Participants who completed and submitted them to the hall managers were included in the research process.

### Data collection tools

The study employed commonly utilized personality and motivation assessments. The "Exercise-2 Behavior Regulation Scale," validated and trustworthy in Turkish by Ersöz et al. [30], was employed to ascertain the participants’ motivations. The "Five Factor Personality Scale," adapted to Turkish culture by Horzum et al. [31], was employed to assess the personality qualities of the subjects.

### Data analysis

For data analysis, bivariate correlation analysis and canonical correlation analysis was used, which tests the structure and dimensionality of the relationship between dependent and independent variable sets in the broadest sense [32]. Canonical correlation analysis is a relationship analysis that supports understanding the relationship between two sets of variables. It supports the reliability of the study data by eliminating the possibility of Type I errors when it comes to studies that usually require multiple regression analyses [33]. Canonical correlation analysis is the most preferred type of analysis in multivariate datasets that have a theoretical basis [34–36]. The basic assumptions of canonical correlation were examined and VIF values were found to be below +2, indicating that there was no multicollinearity [32]. When kurtosis and skewness values were analyzed (Table 1), it was determined that the data showed a normal distribution [37]. In the canonical correlation analysis, the Bartlett Hypothesis was established and the H_0_ hypothesis was defined as "the established canonical correlations are insignificant" and the H_1_ hypothesis as "at least one of the established canonical correlations is significant". Canonical correlation analysis was calculated using IBM SPSS 25.0 software. The results of the canonical correlation analysis of the study were examined and the statistical significance of the generated functions was discussed. In light of the literature review, the "Wilks’ Lambda (λ)" value (Table 3), which is the most widely used, was used in the study [32]. As a result of the analysis, canonical coefficients and correlations, variances, and U-V tables and figures are included for each analysis. For canonical correlation analysis, personality sub-dimensions which is independent variable (variable set V: extraversion, openness to experience, self-control, conscientiousness, and neuroticism) and motivation sub-dimensions which is dependent variable (variable set U: internal regulation, introspective regulation, external regulation, and amotivation) were created.

**Table 1:** Descriptive statistics of the data set.

Variables	Mean	S.d	Kurtosis	Skewness	Variables	Mean	S.d	Kurtosis	Skewness
X1	2.81	0.85	-.688	.294	Y1	7.95	1.94	-.237	1.268
X2	2.06	1.02	-.066	-.497	Y2	6.23	1.24	.055	2.286
X3	0.85	0.84	.679	-.665	Y3	7.58	1.87	-.454	-.382
X4	0.88	0.94	.820	-.372	Y4	5.75	1.78	-.006	-.135
					Y5	7.20	1.78	-.340	.037

## Results and discussion

Table 2 presents correlation analysis and descriptive statistical analysis. In terms of bivariate correlations between participants’ motivation levels and personality traits, internal regulation, external regulation, amotivation, and personality traits exhibited a comparable positive correlation strength. The amotivation dimension showed a weak correlation with the self-control personality trait and did not have a significant relationship with other dimensions. The conscientiousness personality trait showed a weak negative correlation with extraversion and self-control personality traits.

**Table 2:** Descriptive statistics and bivariate correlation of motivation and personality traits.

	M (SD)	1	2	3	4	5	6	7	8	9
**Internal regulation**	2.81 (0.84)	1								
**(1)**										
**Introspective regulation**	2.05 (1.01)	,449**	1							
**(2)**		,000								
**External regulation**	0.86 (0.84)	-,285**	,144**	1						
**(3)**		,000	,006							
**Amotivation**	0.88 (0.93)	-,394**	,020	,641**	1					
**(4)**		,000	,710	,000						
**Extraversion**	7.95 (1.82)	,146**	-,037	-,247**	-,200**	1				
**(5)**		,006	,487	,000	,000					
**Openness to experience**	7.18 (1.77)	,268**	,020	-,253**	-,228**	,218**	1			
**(6)**		,000	,704	,000	,000	,000				
**Self-control**	7.60 (1.84)	,333**	,134*	-,191**	-,247**	,426**	,290**	1		
**(7)**		,000	,011	,000	,000	,000	,000			
**Conscientiousness**	6.22 (1.20)	,016	-,070	,016	-,069	-,220**	,027	-,218**	1	
**(8)**		,761	,186	,761	,195	,000	,615	,000		
**Neuroticism**	5.72 (1.74)	-,206**	-,056	,194**	,200**	-,181**	-,146**	-,163**	,020	1
**(9)**		,000	,289	,000	,000	,001	,005	,002	,705	

*p<0.05

**. p< 0.01

**Fig 1:**
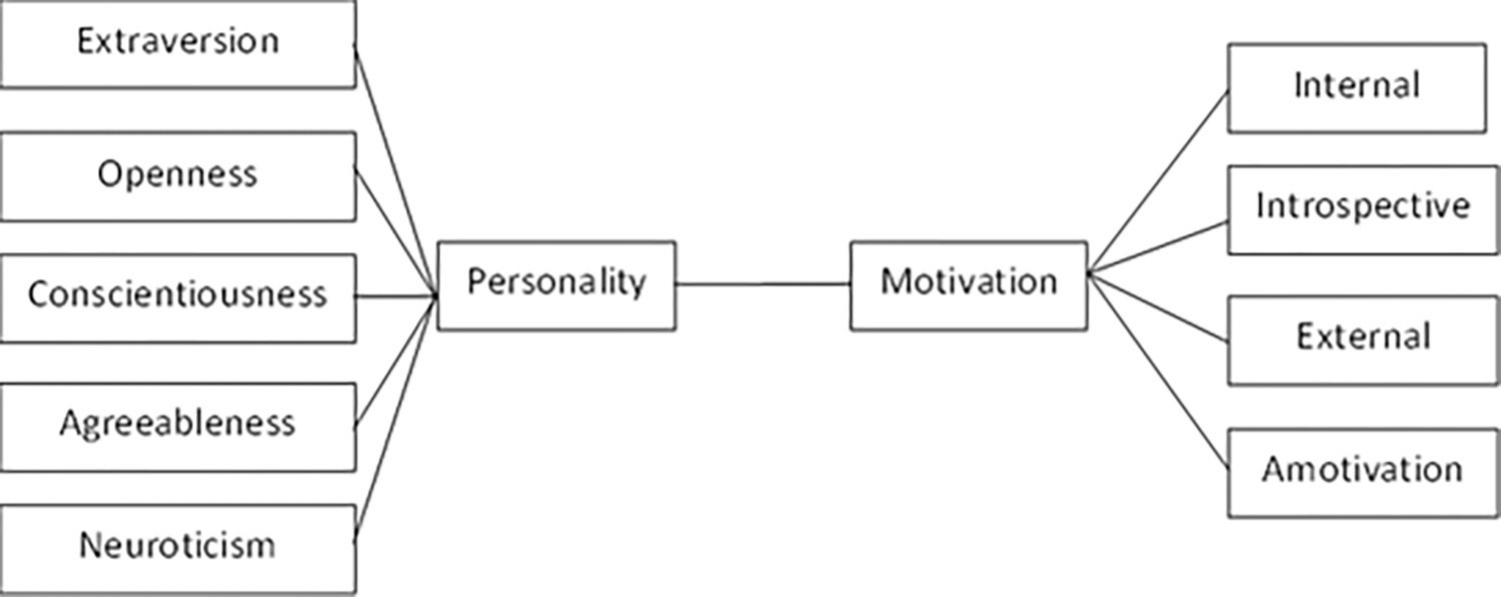
Canonical correlation analysis.

In this study, two different data sets were used to examine the relationship between the personality and motivation structures of the participants. SPSS package software was used for this analysis. The first set (U variable set) includes the participants’ motivational components of internal regulation, introspective regulation, external regulation, and amotivation. The second set (V variable set) included the participants’ personality components of extraversion, openness to experience, self-control, conscientiousness, and neuroticism (Fig 1).

When canonical correlation values were evaluated at the significance level, U1-V1 and U2-V2 canonical correlation values were found to be statistically significant (Wilks λ = 0.733; 0.938; p<0.05), while U3-V3 and U4-V4 canonical correlation values were not statistically significant (p>0.05) (Table 3). Since the first and second canonical functions between the two sets of variables were found to be statistically significant, the first and second canonical function values were interpreted in the evaluation phase. Accordingly, when the canonical correlation values were analyzed, it was determined that the first canonical correlation value was 0.468 and the common variance in the first function of motivation and personality sets was 21.9%. In the second function, the canonical correlation value was 0.201 and the common variance was 4% (Table 3).

**Table 3:** Canonical correlation coefficients and multivariate significance tests.

	Canonical Correlation Coefficient	Square of Canonical Correlation	Eigen value	Wilks’s Lambda	Chi-sq	Df	p
U1-V1	.468	.219	.280	.733	109.565	20.000	**.000**
U2-V2	.201	.040	.042	.938	22.568	12.000	**.032**
U3-V3	.141	.020	.020	.978	7.979	6.000	.240
U4-V4	.052	.003	.003	.997	.939	2.000	.625

When the relationships between the U_1_ variable set were analyzed, it was found that they varied between -.698 and .299 for the first canonical function, and between -.890 and .306 for the second canonical correlation function (Tables 4 and 5). In terms of canonical correlation coefficient, for the first canonical function, internal regulation (x1) sub-dimension and for the second canonical function, external regulation (x3) sub-dimensions were found to be the highest factors. In terms of canonical and cross-loadings, while the highest value for the first canonical function remained the same, the highest value for the second canonical function was found to be introspective regulation (x2).

**Table 4:** Standardized and raw canonical correlation coefficients for the U set of variables.

Variables	Standardized				Raw			
	1	2	3	4	1	2	3	4
**X1**	-.698	-.359	-.133	-.988	-.831	-.426	-.158	-1.175
**X2**	.096	-.432	.790	.761	.096	-.429	.786	.757
**X3**	.283	-.890	-.958	-.012	.338	-1.064	-1.145	-.014
**X4**	.299	.306	.897	-.932	.322	.331	.968	-1.006

**Table 5:** Canonical charges and cross-loads for the U variable set.

Variables	Canonical charges				Cross-Loads			
	1	2	3	4	1	2	3	4
**X1**	-.854	-.419	.141	-.276	-.399	-.084	.020	-.014
**X2**	-.171	-.714	.610	.298	-.080	-.144	.086	.015
**X3**	.687	-.654	-.231	-.217	.322	-.132	-.033	-.011
**X4**	.757	-.131	.352	-.535	.354	-.026	.049	-.028

Table 6 shows the standardized and raw canonical correlation coefficient values of the V1 variable set. It was found that the first canonical function values varied between -.534 and .364, and the second canonical correlation function, which was significant, values, varied between -.902 and .906. In terms of canonical correlation coefficient, for the first canonical function, self-control (y3) sub-dimension, and for the second canonical function, extraversion (y1) sub-dimensions were found to be the highest factors. Table 7 shows the canonical charges and cross-loadings. It was determined that the highest value did not change in terms of both canonical functions.

**Table 6:** Standardized and raw canonical correlation coefficients for the V set of variables.

Variables	Standardized				Raw			
	1	2	3	4	1	2	3	4
**Y1**	-.161	.906	-.217	.365	-.088	.497	-.119	.200
**Y2**	-.445	.315	.187	-.847	-.250	.177	.105	-.477
**Y3**	-.534	-.902	-.046	.112	-.290	-.490	-.025	.061
**Y4**	-.220	-.060	-1.016	.032	-.184	.050	-.849	.027
**Y5**	.364	-.001	-.161	-.588	.209	-.001	-.092	-.338

**Table 7:** Canonical charges and cross-loads for the V variable set.

Variables	Canonical charges				Cross-Loads			
	1	2	3	4	1	2	3	4
**Y1**	-.503	.604	.057	.328	-.235	.122	.008	.017
**Y2**	-.694	.249	.123	-.648	-.325	.050	.017	-.033
**Y3**	-.743	-.411	.164	.111	-.348	-.083	.023	.006
**Y4**	-.072	-.054	-.956	-.108	-.034	-.011	-.135	-.006
**Y5**	.541	-.065	-.162	-.548	.253	-.013	-.023	-.028

**Fig 2:**
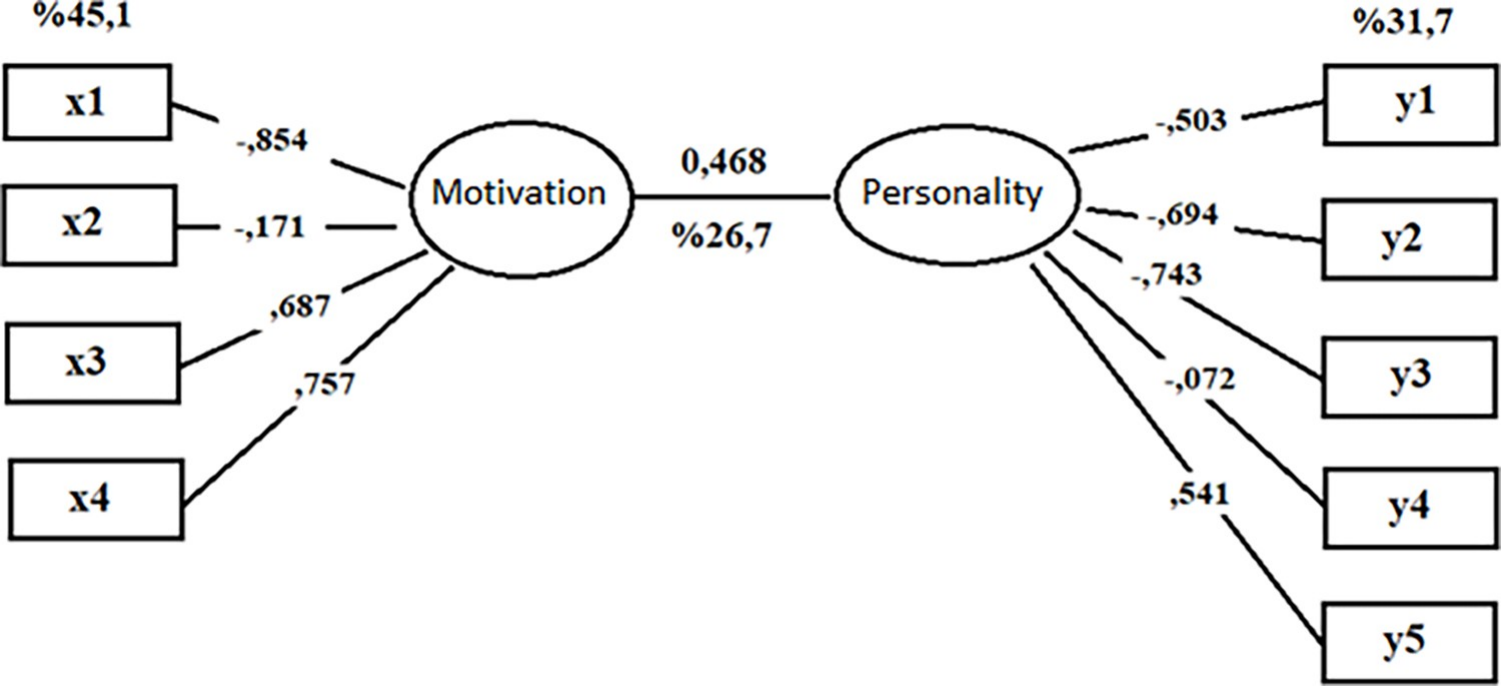
Canonical correlation for U1-V1 function.

**Fig 3:**
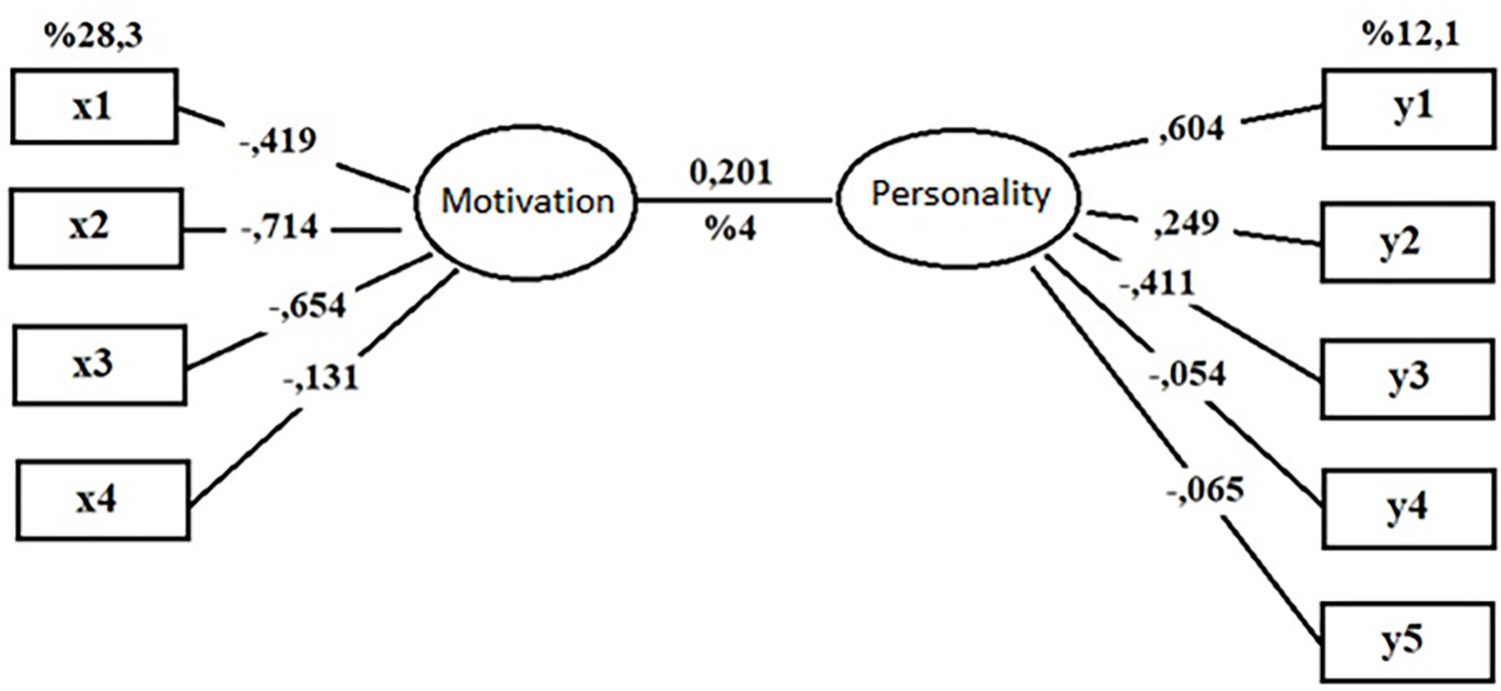
Canonical correlation for U2-V2 function.

The variance values explained by the canonical variables are presented in Table 8. Accordingly, the representations and explanations of the function created between U_1_-V_1_ (Fig 2) and U_2_-V_2_ (Fig 3) are given below.

**Table 8:** Explained variance ratios for U-V canonical functions.

Canonical Variables	Set 1 by Self	Set 1 by Set 2	Set 2 by Self	Set 2 by Set 1
**1**	.451	**.099**	.317	**.069**
**2**	.283	.011	.121	.005
**3**	.142	.003	.197	.004
**4**	.125	.000	.170	.000

Fig 2 shows the first canonical function values generated between the U-V variable sets. It can be stated that Function 1 explains approximately 26.7% of the canonical correlation between the U_1_ data set, motivation (x1: internal regulation, x2: introspective regulation, x3: external regulation, x4: amotivation), and the V_2_ data set, personality (y1: extraversion, y2: openness to experience, y3: self-control, y4: conscientiousness, y5: neuroticism). The U_1_ dataset explains approximately 45.1% of the total variance and the V_1_ dataset explains approximately 31.7% of the total variance. In the canonical correlation analysis for Function 1, the most influential factors in motivation variable loadings were the sub-dimensions of internal regulation (load: -.854), amotivation (load: .757), external regulation (load: .687) and introspective regulation (load: -.171), respectively. The most influential factors in the canonical loadings of the personality variable set were the sub-dimensions of self-control (load: -.743) openness to experience (load: -.694), neuroticism (load: .541), extraversion (load: -.503), and conscientiousness (load: -.072), respectively (Fig 2). The first canonical function can be expressed as follows:

U_1_ = -0.854x1-0.171x2+0.687x3+0.757x4

V_1_ = -0.503y1-0.694y2-0.743y3-0.072y4+0.541y5

Fig 3 shows the second canonical function values generated between the variable sets. For the second function, which was found to be statistically significant, it can be stated that it explains approximately 4% of the canonical relationship. The U_2_ dataset explains approximately 28.3% of the total variance and the V_2_ dataset explains approximately 12.1% of the total variance. As can be seen in Fig 3, for the U_2_-V_2_ function, the most influential factors in motivation variable loadings were the sub-dimensions of internalizing regulation (load: -.714), external regulation (load: -.654), introspective regulation (load: -.419), and amotivation (load: -.131), respectively. The most influential factors in the canonical loadings of the personality variable set were the sub-dimensions of extraversion (load: .604), self-control (load: -.411), openness to experience (load: .249), neuroticism (load: -.065), and conscientiousness (load: -.054), respectively. The second canonical function can be expressed as follows:

U_2_ = -0.419x1-0.714x2-0.654x3-0.131x4

V_2_ = 0.604y1+0.249y2-0.411y3-0.054y4-0.65y5

Canonical correlation analyses are among the analyses applicable to data sets belonging to two different variables [38]. There is a relationship between personality traits and motivation that determines one’s personality [39]. Considering the outcomes obtained from the data, it was determined that the personality traits of self-control and extraversion and the motivation types of internal regulation and external regulation were among the significant variables for leisure participants. In the first canonical function, self-control and internal regulation are in the unidirectional relationship, indicating that they support each other. Terracciano et al, [40] revealed in their study that differences arising from personality become most important during more challenging physical activities. When the types of motivation were examined, it was determined that it targets intrinsic motivation among leisure goals [41]. It can be considered as an expected result that individuals with high intrinsic motivation will also have a high perspective on the experience offered by leisure time. In the second canonical function, it was observed that leisure time participants with extraverted personality traits had a negative relationship with extrinsic motivation type. Considering that leisure time is an individual-specific experience [42], the general characteristics of extraverted individuals include sociability, mobility, talkativeness, and assertiveness [43], while extrinsic motivation is the extent to which an individual is willing to do something and the pressure, they feel about it [44]. However, the fact that the control over the reward or the criticism and praise given to the individual comes from an external source causes the individual’s intrinsic motivation to weaken [45]. In this case, it can be considered that for an extroverted individual, an extrinsic source of motivation in leisure time activities would not have a positive meaning for the individual.

Considering the literature, there are different studies examining the relationship between personality and motivation. Zorlu et al. [46] examined the relationship between personality traits and sports achievement motivation of athletes in athletics and found that there is a strong relationship between personality traits and sports achievement motivation of athletes involved in athletics. Modiri [47] examined the relationship between music-teaching students’ piano lesson motivation and personality traits and concluded that there was no relationship between music-teaching students’ piano lesson motivation and personality traits. Turhan [48] found that the achievement motivation of professional football players with the personality trait of "novelty seeking" was higher than that of football players without this personality trait, while the achievement motivation of football players with the personality trait of "harm avoidance" was lower than that of football players without this personality trait. Lonel et al [49], in their study on rock climbing athletes, stated that their results indicated that personality traits affect rock climbing performance in both sport climbing and rock climbing. Kekalainen et al. [50], in their study titled The Effect of Personality and Individual Differences on Physical and Cognitive Activity, stated that personality traits mostly have empty moderating effects on physical and cognitive results. They stated that personality traits do not have much effect on physical functionality and cognitive functionality results. Çiftçi [51] examined the relationship between participation motivations and personality traits of individuals participating in sports and found a positive low-level relationship between the health sub-dimension of exercise motivation and all personality traits, a positive low-level relationship between the body appearance sub-dimension and extraversion and openness to development personality traits, and a positive low-level relationship between the skill development sub-dimension and all personality traits except neuroticism. Piepiora et al. [52], in his study on the personality traits and sports levels of athletes, explained that the level of neuroticism, one of the personality sub-dimensions, significantly affects athletic performance, and that lower levels are characteristic of professional athletes and contribute to better emotional balance and stress management. In contrast, Klatt et.al [53] in their study on personality and emotional states in elite beach volleyball players revealed that personality and emotional regulation were not related to the actual performance level. Keerthika and Punithavathi [54], in their study on athletes, revealed that there is no significant relationship between athletes’ personalities and motivation types. Piepiora et al. [55] In their study on Polish mountain climbers, they revealed a significant difference in the personalities of Polish Alpine and Polish Himalayan Mountain climbers in the compatibility dimension only among women. Vlašić and Ivanišević [56], in their study examining the relationship between personality traits and achievement motivation of selected sports activities, concluded that personality traits did not have a dependent relationship with the choice of individual or team sport, playing sport professionally or recreationally, and achievement motivation. Kekäläinen et al. [57], in their study on the determinants of personality, motivation, and social cognition of leisure-time physical activity, found that motivation directly predicted and was related to leisure-time physical activity. In another area, the results of another study conducted by Achakul and Yolles in a manufacturing company operating in Thailand also show that there is a significant and strong relationship between all personality traits and the importance given to intrinsic and extrinsic motivation [58].

## Conclusion

Studies show once again how valuable intrinsic motivation sources are for individuals, and personality traits such as self-control and extraversion support this situation. By increasing the size of the sample group, the results can be observed and evaluated in large-scale studies. By changing the sample group of the current study, the literature can be supported by determining the personality traits and motivation types of individuals who do not participate in physical activity in their leisure time. Thus, by encouraging individuals to participate in physical activity, they can utilize their leisure time more efficiently and effectively. In addition, since it is thought that the society in which one lives has an effect on the formation of personality traits, cross-cultural studies can be conducted with other countries.
